# HMGA2 enhances 5-fluorouracil chemoresistance in colorectal cancer via the Dvl2/Wnt pathway

**DOI:** 10.18632/oncotarget.24133

**Published:** 2018-01-10

**Authors:** Xi Xu, Yunfeng Wang, Hong Deng, Chungang Liu, Jingjing Wu, Maode Lai

**Affiliations:** ^1^ Department of Pathology, School of Medicine, Zhejiang University, Hangzhou, Zhejiang, 310058, China; ^2^ Key Laboratory of Disease Proteomics of Zhejiang Province, Hangzhou, Zhejiang, 310058, China; ^3^ College of Bioinformatics Science and Technology, Harbin Medical University, Harbin, Heilongjiang, 150081, China; ^4^ Center of Biological Therapy, Institute of Pathology and Southwest Cancer Center, Southwest Hospital, Third Military Medical University, Chongqing, 400038, China

**Keywords:** HMGA2, Dvl2, 5-FU, chemoresistance, colorectal cancer

## Abstract

Drug resistance is one of the main hurdles to overcome for the improvement of cancer patient survival. However, the underlying mechanisms remain largely unknown, and therapeutic options are limited. Here, we demonstrate a strong correlation between HMGA2 expression and chemosensitivity to 5-fluorouracil (5-FU), a widely used first-line systemic chemotherapy regimen for colorectal cancer (CRC) patients. Overexpression of HMGA2 enhances chemoresistance to 5-FU of CRC both *in vitro* and *in vivo*. Further experiments indicate that HMGA2 directly binds to the promoter of *Dvl2* and induces its transcription, which leads to increased activation of the Wnt/β-catenin pathway. Taken together, our data suggest that HMGA2 enhances the chemoresistance to 5-FU in CRC via activating the Dvl2/Wnt pathway. Therefore, HMGA2 may serve as a predictive biomarker and a potential therapeutic target in CRC.

## INTRODUCTION

Colorectal cancer is the third most common type of tumor worldwide. In the USA, more than 1.3 million new cases of CRC are diagnosed every year [1]. Various chemotherapeutic drugs have been used effectively for quite some time; however, 5-FU remains the most widely employed chemotherapeutic agent in CRC treatment [2]. Although 5-FU-based chemotherapy has improved response rates to greater 50% and achieves a median survival rate of up to 2 years [3, 4], failure of this treatment in > 90% of patients is due to drug resistance.

HMGA2 belongs to the non-histone chromosomal high-mobility group (HMG) family and is located at 12q 13-15. Human HMGA2 consists of five exons and four introns. The first three exons encode three DNA-binding domains responsible for preferential binding of HMGA2 to adenine-thymine (AT)-rich minor grooves of nuclear B-form DNA via its “AT hooks” [5]. Hence, HMGA2 functions as an architectural transcription factor by altering target chromatin structure and through protein-DNA and protein-protein interactions [6–8]. HMGA2 is commonly overexpressed in numerous malignant cancers and is associated with increased invasiveness, stemness and a poor prognosis due to mediating downstream pathways, such as the TGFβ [9] and Akt [10] pathways.

In this study, we explored the roles of HMGA2 in maintaining the 5-FU chemoresistance of CRC *in vitro* and *in vivo*. Moreover, we identified a novel target gene of HMGA2 that may be involved in malignant phenotypes. Luciferase and chromatin immunoprecipitation (ChIP) assays revealed that HMGA2 bound directly to the promoter of the Dvl2 and thereby up-regulating β-catenin/TCF transcriptional activity. In addition, we found that HMGA2 and Dvl2 expressions were strongly positively correlated in CRC specimens. Collectively, our findings suggested that HMGA2 promoted the 5-FU resistance of CRC via activating the Dvl2/Wnt signaling pathway. Thus, HMGA2 could be a potential target for addressing drug resistance in CRC.

## RESULTS

### Increased expressions of HMGA2 in the non-responder group of FOLFOX regimen in CRC

**Figure 1:**
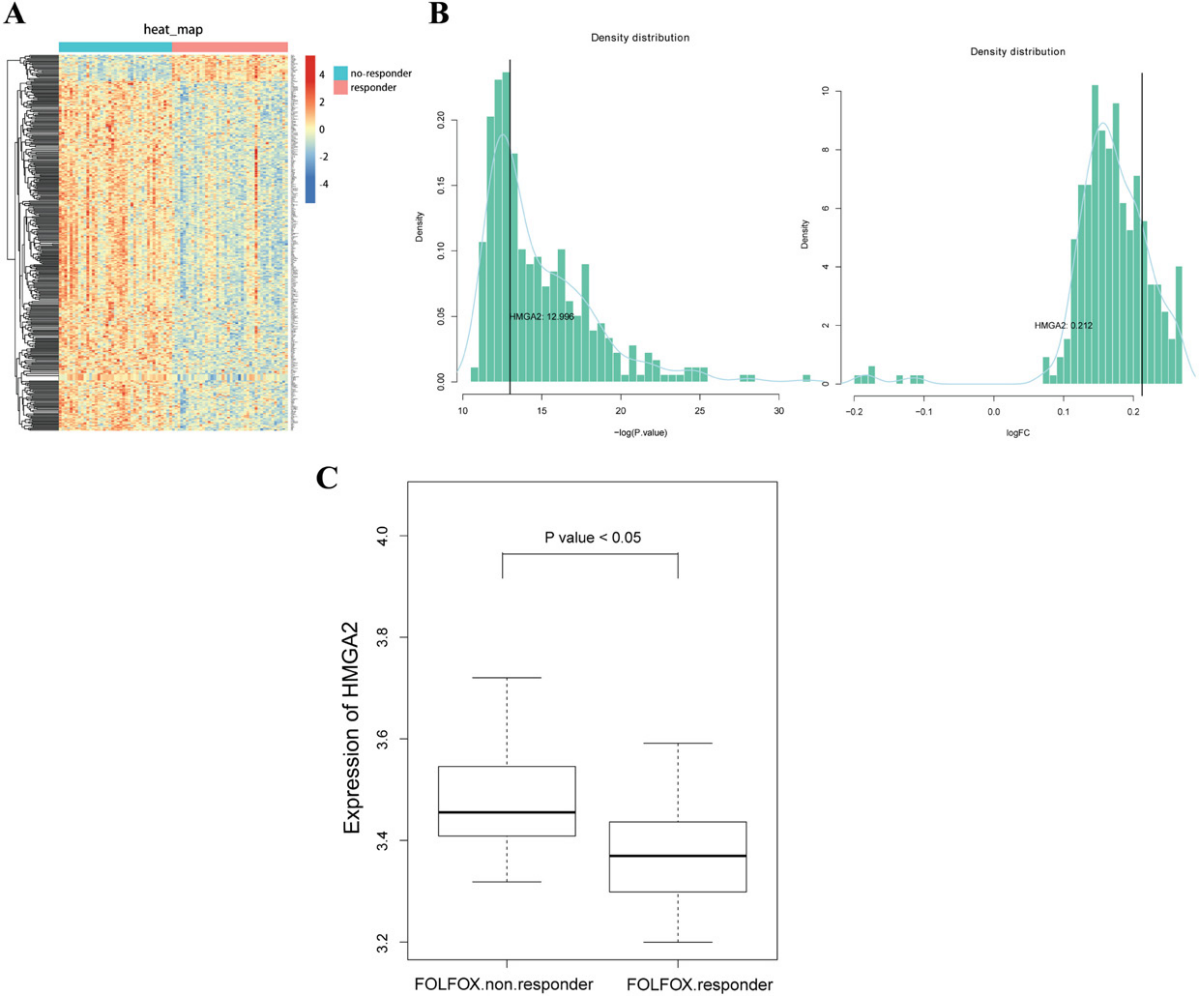
Increased expression of HMGA2 in the non-responder group of FOLFOX regimen in CRC (**A**) The expression levels of differential expressed genes between responder and non-responder samples in GSE28702 datasets, in which responder samples were used as control. (**B**) Density distributions of log-transformed *P* values and fold changes from all the differential expressed genes. The log-transformed *P* value and fold change of gene HMGA2 were 12.996 and 0.212, respectively. Therefore, HMGA2 was significantly upregulated in non-responder samples. (**C**) Boxplot of HMGA2 expressions in two groups. The mean value of HMGA2 expression was significantly higher in the non-responder group.

First, we sought to assess the potential role of HMGA2 in CRC chemoresistance. Thus, we searched microarray data related to CRC resistance to FOLFOX (oxaliplatin, 5-FU, and leucovorin) regimen in the Gene Expression Omnibus (GEO) database and selected GSE28702 [11]. A heat map indicated that HMGA2 belonged to a set of genes that were upregulated in the non-responder group compared with the responder group (Figure 1A and 1B). As shown in Figure 1C, high levels of HMGA2 mRNA expression were seen in the non-responder group compared with that in the responder group (*P* < 0.05). These observations indicated that HMGA2 may be associated with 5-FU chemoresistance in CRC.

### HMGA2 enhances chemoresistance against 5-FU in CRC cells *in vitro*

**Figure 2:**
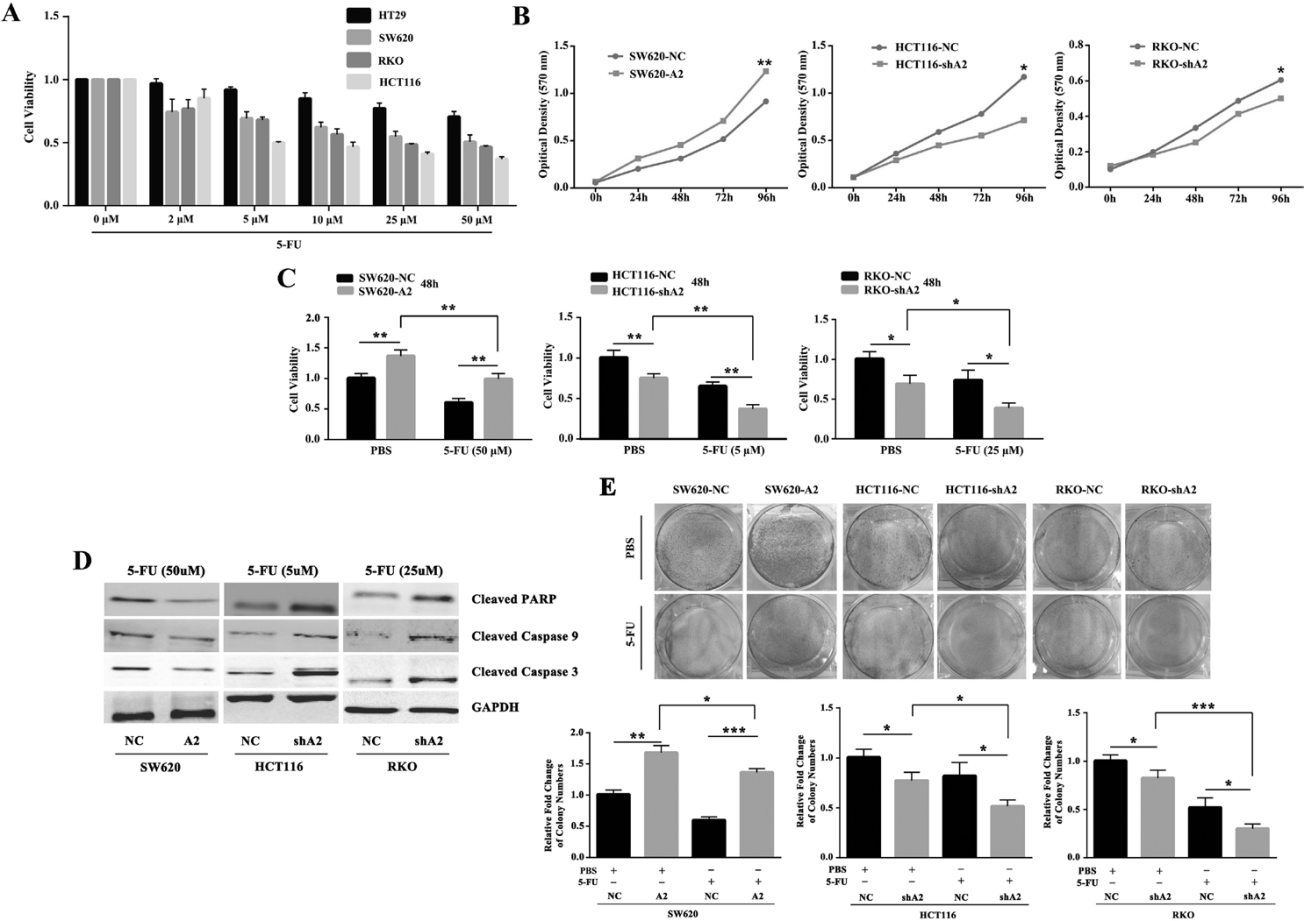
HMGA2 enhances chemoresistance against 5-FU in CRC cells *in vitro* (**A**) Colorectal cancer cells showed differential responses to 5-FU treatment. (**B**) MTT assay showed HMGA2 promoted proliferation. (**C**) MTT assays were performed to examine cell viability in SW620-NC/SW620-A2, HCT116-NC/HCT116-shA2 and RKO-NC/RKO-shA2 cells after treatment with the indicated doses of 5-FU or PBS. (**D**) Western blot analysis of Cleaved-PARP, Cleaved-Caspase-9 and Cleaved-Caspase-3 expressions in the indicated CRC cells following the treatment of 5-FU. GAPDH was used as an internal control. (**E**) Colony formation assays were performed in SW620-NC/SW620-A2, HCT116-NC/HCT116-shA2 and RKO-NC/RKO-shA2 cells after treatment with the indicated doses of 5-FU or PBS. The data were presented as the mean ± S.D. of triplicate experiments. ^*^P < 0.05, ^**^P < 0.01, ^***^P < 0.001.

To determine the effect of 5-FU in CRC *in vitro*, we performed MTT assays in a panel of CRC cell lines. Our results indicated that 5-FU decreased cell viability in a dose-dependent manner, especially in SW620, HCT116 and RKO cells (Figure 2A). To examine whether HMGA2 contributes to 5-FU resistance, we overexpressed HMGA2 in SW620 cells (SW620-A2) and knocked it down in HCT116 (HCT116-shA2) and RKO cells (RKO-shA2) as described previously [12]. As shown in Figure 2B, HMGA2 could promote proliferation in CRC cells. Overexpression of HMGA2 partially remitted the attenuated proliferation and increased apoptosis in the SW620 cells with 5-FU treament. Consistently, knockdown of HMGA2 affected the proliferation and apoptosis in a similar way in HCT116 and RKO cells. (Figure 2C–2E). Taken together, these results indicated that overexpression of HMGA2 protected CRC cells from 5-FU *in vitro*.

### HMGA2 induces 5-FU chemoresistance of CRC *in vivo*

**Figure 3:**
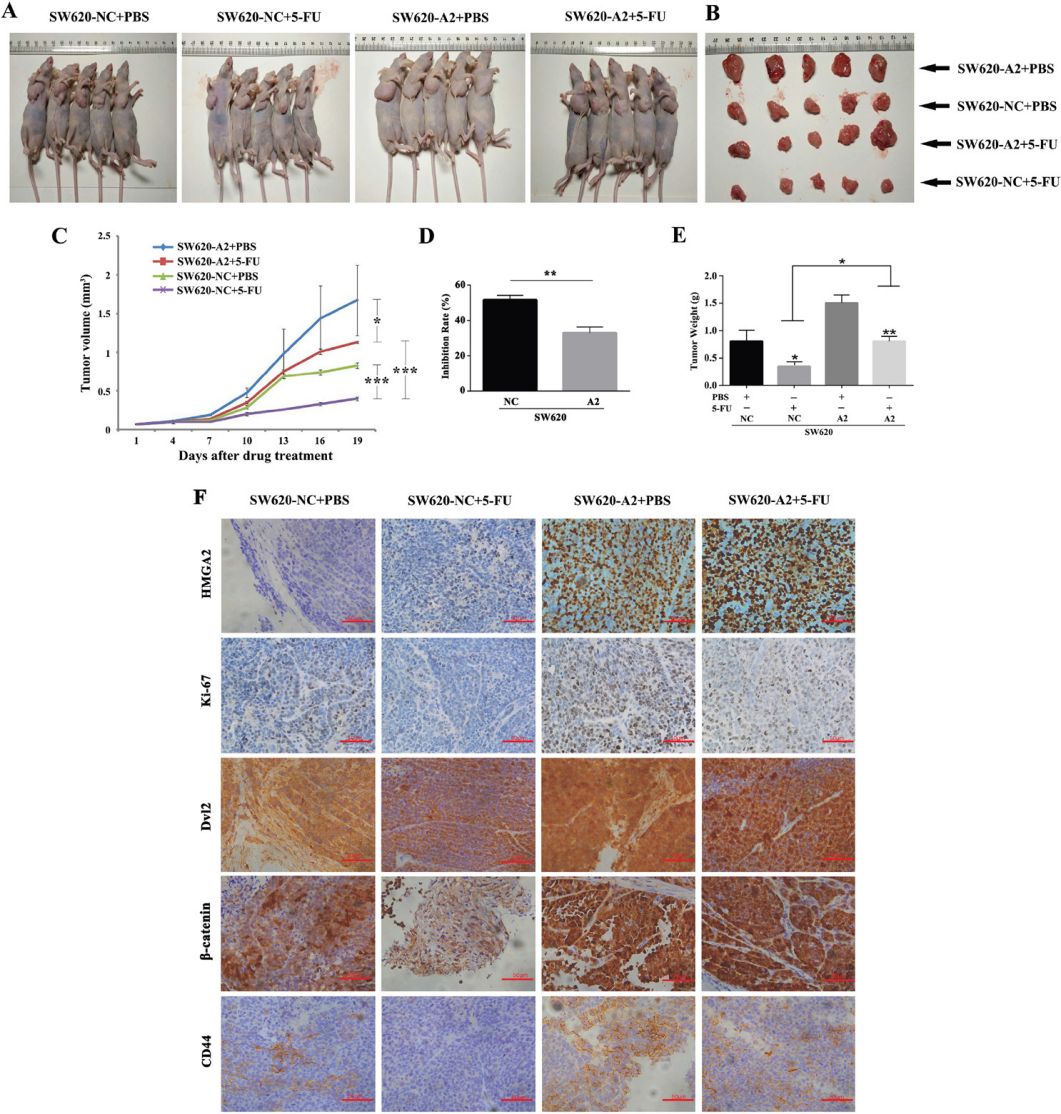
HMGA2 induces 5-FU chemoresistance of CRC *in vivo* (**A** and **B**) Images of mice and tumors were taken at the end of the study on the same scale. (**C**) Xenograft tumor growth curves from day 1 to day 19 after 5-FU treatment. Inhibition rate (**D**) and tumor weights (**E**) at the end of the study were shown. (**F**) Representative images of HMGA2, Ki67, Dvl2, β-catenin and CD44 staining of tumors were shown. The data were presented as the mean ± S.D. ^*^P < 0.05, ^**^P < 0.01, ^***^P < 0.001.

To determine whether HMGA2 increased chemoresistance to 5-FU *in vivo*, we used a colorectal cancer xenograft model. 5 mice were used in each of group. Briefly, tumor growth curves showed that 5-FU inhibited tumor growth in both the HMGA2-overexpressing and vector groups. Furthermore, HMGA2 promoted tumor growth compared with the vector control, and group with HMGA2 overexpression exhibited marked chemoresistance in the response to 5-FU treatment (Figure 3A and 3C). As shown in Figure 3D, the inhibition rate of SW620-NC group was significantly higher than that of SW620-A2 group following the treatment of 5-FU. The mean tumor size (Figure 3B) and weight (Figure 3E) in the HMGA2-transfected group were significantly higher than those in the control group after 5-FU treatment. As shown in Figure 3F, immunoreactivity for HMGA2 was significantly higher in HMGA2-transfected group than in controls. Moreover, immunohistochemistry staining showed that HMGA2-overexpressing tumors contained more Ki67 (+) cells under the condition of 5-FU treatment compared with the vector controls (Figure 3F). Similar results were obtained from Dvl2, β-catenin, and CD44 staining (Figure 3F). Collectively, these results indicated that HMGA2 regulated chemoresistance to 5-FU of CRC *in vivo*.

### HMGA2 regulates various cellular processes, including the Wnt pathway

**Figure 4:**
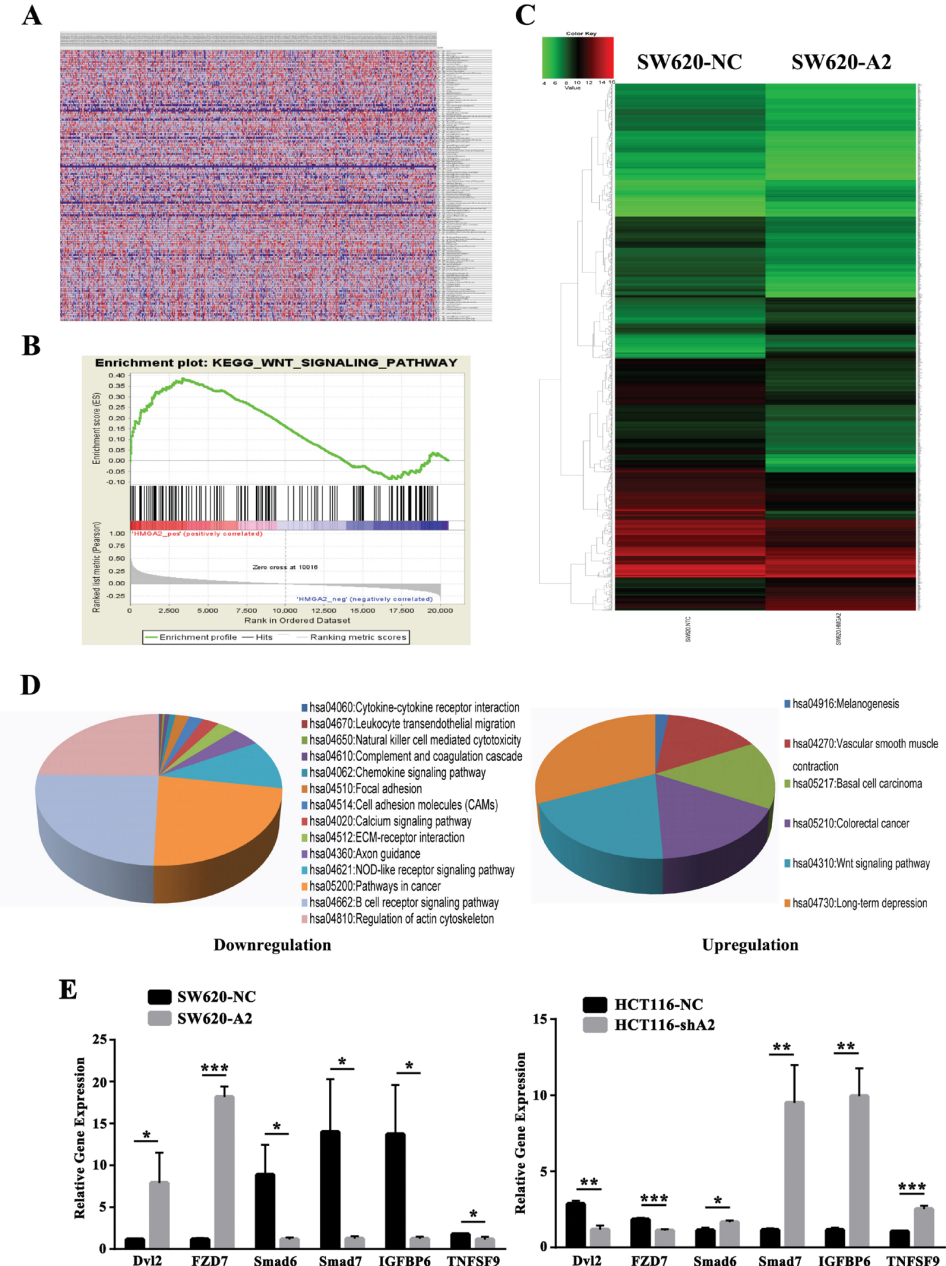
Bioinformatics analysis for cellular signaling regulated by HMGA2 (**A** and **B**) Gene set enrichment analysis based on microarray data from TCGA dataset. (**C**) The whole-genome expression patterns for SW620-NC (left) and SW620-A2 (right) were shown as heat maps. (**D**) Genes for which expression levels were altered (fold change > 2) by HMGA2 were ordered by function-related groups. (**E**) Quantitative RT-PCR analysis of the Dvl2, FZD7, Smad6, Smad7, IGFBP6 and TNFSF9 mRNA expressions in the indicated CRC cells. The data were presented as the mean ± S.D. ^*^P < 0.05, ^**^P < 0.01, ^***^P < 0.001.

To determine the signaling pathways that were potentially regulated by HMGA2 in CRC, we performed GSEA [13, 14] using high-throughput RNA-sequencing data from the TCGA database to examine the mode of action of HMGA2. We observed that HMGA2 was correlated with many oncogenetic gene sets, such as sets related to calcium signaling, Wnt signaling and arachidonic acid metabolism (Supplementary Tables 2 and 3). Among all of the predefined KEGG gene sets, the KEGG Wnt pathway was identified as one of signaling pathways showing the strongest association with HMGA2 expression (NES = 1.51; *P* = 0.037) (Figure 4A and 4B).

We performed the mRNA expression array on SW620-NC and SW620-A2 cells to identify target genes of HMGA2. A total of 1,691 genes were dysregulated after HMGA2 overexpression (Figure 4C). We then focused on genes showing > 2-fold change in expression for functional enrichment analysis using DAVID Bioinformatics Resources 6.7 [15]. Numerous genes involved in cancer-related inflammatory signaling pathways, the Wnt signaling pathway and cell adhesion were characterized (Figure 4D). Microarray and qPCR data were particularly well correlated for six genes that were differentially regulated between the SW620-NC/SW620-A2 or HCT116-NC/HCT116-shA2 groups (Figure 4E), including Wnt pathway-related genes, such as Dvl2 and FZD7. Collectively, these results indicated that HMGA2 was involved in Wnt pathway.

### HMGA2 directly binds to the Dvl2 promoter to activate its transcription

**Figure 5:**
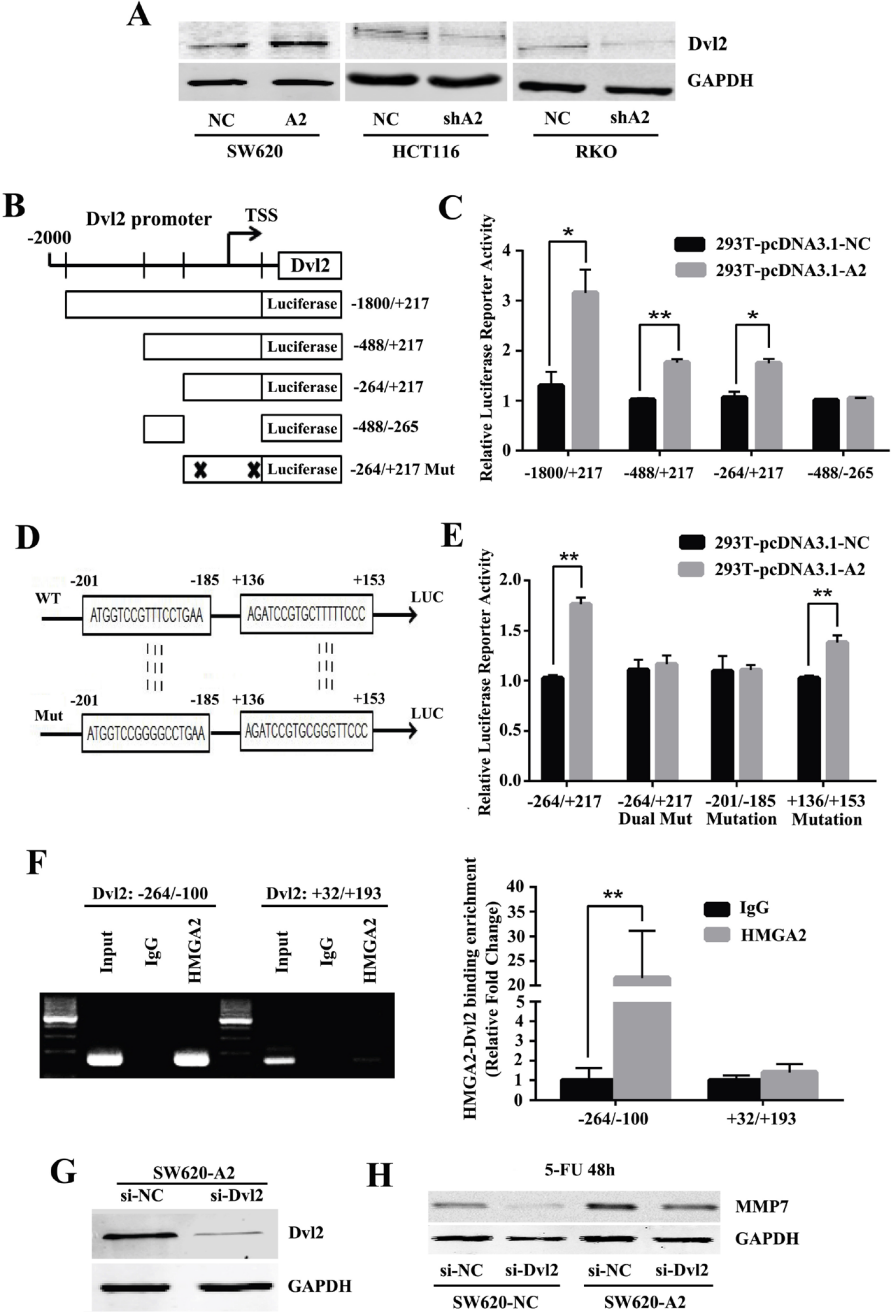
HMGA2 directly binds to the Dvl2 promoter to activate its transcription (**A**) Western blot analysis of Dvl2 expressions in the indicated CRC cells. GAPDH was used as an internal control. (**B**) Schematic presentation of the luciferase plasmids Dvl2-Luc and their truncated derivatives. (**C**) Histobars showed the relative luciferase activity of Dvl2 promoter in HEK293T cells with or without HMGA2 overexpression when transfected with the truncated luciferase plasmids. (**D**) Schematic presentation of the mutated luciferase plasmids. (**E**) Histobars showed the relative luciferase activity of Dvl2 promoter in HEK293T cells with or without HMGA2 overexpression when transfected with the mutant luciferase plasmids. (**F**) ChIP-PCR and ChIP-qPCR assays were performed using specific primer pairs for the Dvl2 promoter. ^*^P < 0.05, ^**^P < 0.01. (**G**) Western blot analysis of Dvl2 protein levels in the SW620-A2 cells transfected with control or Dvl2 siRNA. (**H**) Western blot analysis of MMP7 in SW620-NC and SW620-A2 cells treated with siNC/siDvl2 and 5-FU for 48 h. GAPDH was used as a loading control.

To further investigate the mechanisms by which HMGA2 executed its function, we adopted the ChIP-on-chip assay for target gene prediction. Among the candidate genes, Dvl2, an important oncogene, was identified as one of the potential targets of HMGA2 and selected for further analysis. We conducted western blotting to measure the effect of HMGA2 on endogenous Dvl2 expression. As shown in Figure 5A, Dvl2 was induced by HMGA2 overexpression in SW620 cells, whereas it was reduced following HMGA2 knockdown in HCT116 and RKO cells.

To test whether Dvl2 may be a direct target of HMGA2, we generated luciferase reporter assays to identify the transcriptional pattern of Dvl2 induced by HMGA2 (Figure 5B). Our results demonstrated that the promoter region without –264/+217 sequence exhibited a minimal response to HMGA2 stimulation (Figure 5C), suggesting that the –264/+217 region of Dvl2 was essentially responsible for the function of HMGA2.

Then we analyzed the sequence of the –264/+217 region of the Dvl2 promoter. And two adenine-thymine (AT)-rich sequences were identified, including the Dvl2 promoter regions of –201/–185 and +136/+153. Corresponding point mutants were developed and used for luciferase reporter analysis (Figure 5D). As shown in Figure 5E, mutating these two sequences simultaneously abolished luciferase activity induced by HMGA2, indicating that these presumptive sequences were the direct binding points targeted by HMGA2. Furthermore, by mutating these sequences respectively, we found that sequence from –201 to –185 mainly conferred the transcriptional activity of Dvl2 promoter in response to HMGA2 (Figure 5E). ChIP-PCR and ChIP-qPCR analysis further demonstrated that HMGA2 directly bound to the Dvl2 promoter region of –264/–100 (Figure 5F). Hence, these findings suggested that HMGA2 may transactivate Dvl2 through directly binding to the site from –201 to – 185.

Given the results obtained so far, we next sought to explore whether Dvl2 affects the chemoresistance of CRC cells induced by HMGA2. Thus, we studied the effects of silencing Dvl2 on SW620-NC and SW620-A2 cells. Dvl2 was remarkably inhibited by siRNA, as shown by western blot analysis (Figure 5G). In order to investigate whether HMGA2 influences the effects of Dvl2, MMP7, a downstream target of Dvl2 was used [16]. The results showed that the upregulation of MMP7 expression by HMGA2 was significantly reduced by silencing Dvl2 after 5-FU treatment (Figure 5H). All of these data demonstrated that HMGA2 directly binds to the Dvl2 promoter to activate its transcription.

### HMGA2 modulates the downstream signaling pathway of Dvl2

**Figure 6:**
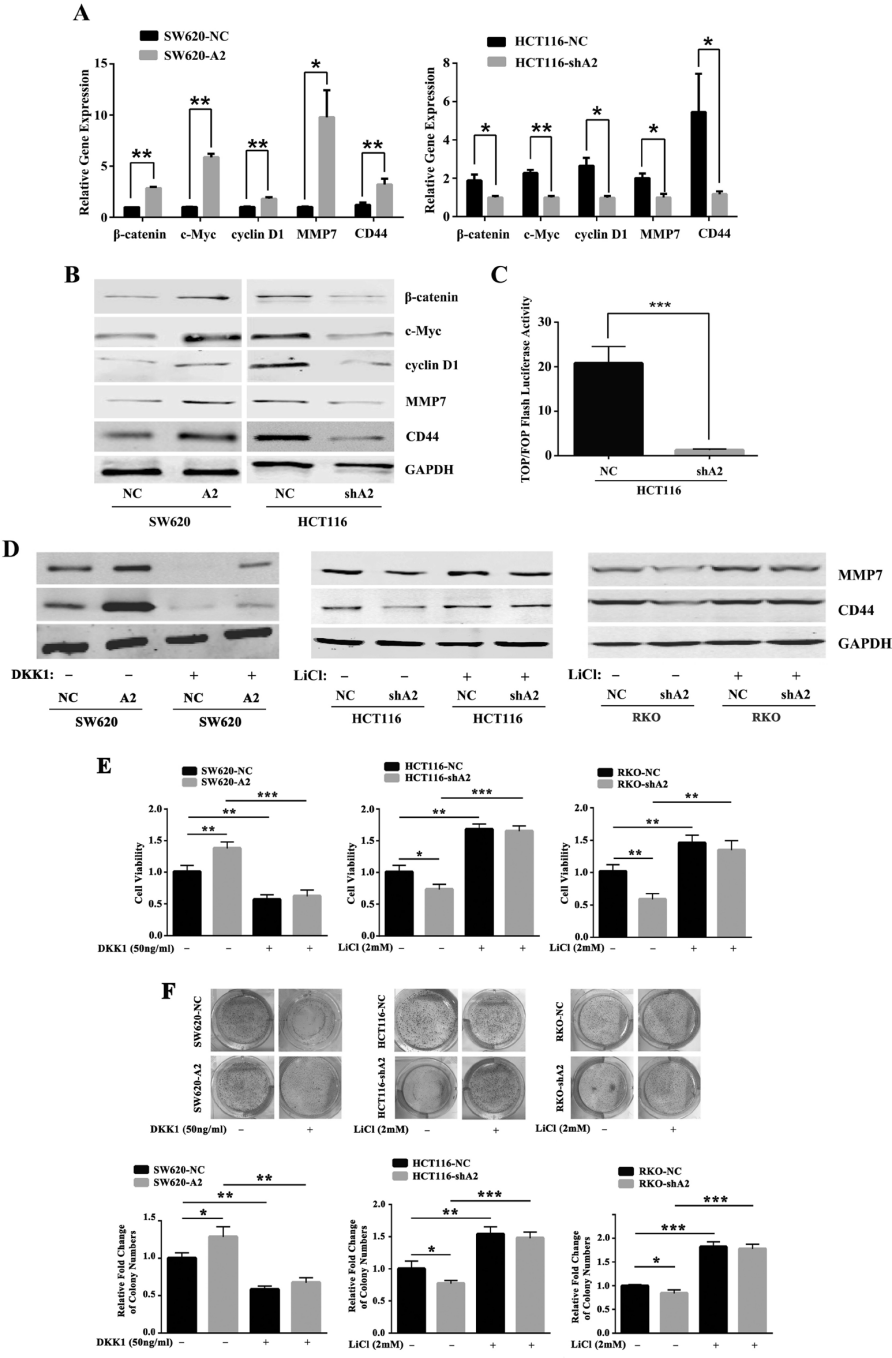
HMGA2 modulates the downstream signaling pathway of Dvl2 Quantitative RT-PCR (**A**) and Western blot analysis (**B**) of the β-catenin, c-Myc, cyclin D1, MMP7 and CD44 expressions in the indicated cells. GAPDH was used as an internal control. ^*^P < 0.05, ^**^P < 0.01. (**C**) Luciferase activity showed that silencing of HMGA2 resulted in a significant decrease in the ratio of TOP Flash to FOP Flash activity in HCT116 cell line. ^***^P < 0.001. (**D**) Western blot analysis of MMP7 and CD44 in SW620-NC/SW620-A2 cells treated with or without DKK1 and in HCT116-NC/HCT116-shA2 and RKO-NC/RKO-shA2 cells treated with or without LiCl. GAPDH was used as an internal control. MTT (**E**) and colony formation assays (**F**) were performed in SW620-NC/SW620-A2 cells treated with or without DKK1 and in HCT116-NC/HCT116-shA2 and RKO-NC/RKO-shA2 cells treated with or without LiCl. The data were presented as the mean ± S.D. of triplicate experiments. ^*^P < 0.05, ^**^P < 0.01, ^***^P < 0.001.

It is well-known that Dvl2 is a key mediator of Wnt/β-catenin. To confirm the relationship between HMGA2 and Wnt signaling, we performed qRT-PCR and western blotting to test whether HMGA2 could activate Wnt signaling. As shown in Figure 6A and 6B, HMGA2 significantly upregulated the expressions of β-catenin, c-Myc, cyclin D1, MMP7 and CD44 compared with the control group. In addition, TOP/FOP Flash luciferase assay showed that HMGA2 knockdown consistently and significantly decreased the transcriptional activity of the canonical Wnt reporter (Figure 6C).

Then we used DKK1 to inhibit the Wnt signaling pathway in SW620-A2 and SW620-NC cells. We found that the treatment of DKK1 abolished the induction of MMP7 and CD44 by HMGA2 overexpression (Figure 6D). Conversely, we used LiCl, a GSK3β inhibitor, to activate the Wnt signaling pathway in HMGA2-knockdown cells (HCT116-shA2, RKO-shA2) and their corresponding controls (HCT116-NC, RKO-NC). The results confirmed that Wnt signaling genes MMP7 and CD44 were rescued after the treatment of LiCl in HMGA2-knockdown cells compared with control cells (Figure 6D), which firmly supported the regulation of Wnt signaling by HMGA2 in CRC. Consistent with this, cell viability (Figure 6E) and plate colony formation assays (Figure 6F) indicated that HMGA2-mediated proliferation in CRC cells was Wnt signaling-dependent.

### Dvl2 expression is positively correlates with HMGA2 expression in human CRC tissues

**Figure 7:**
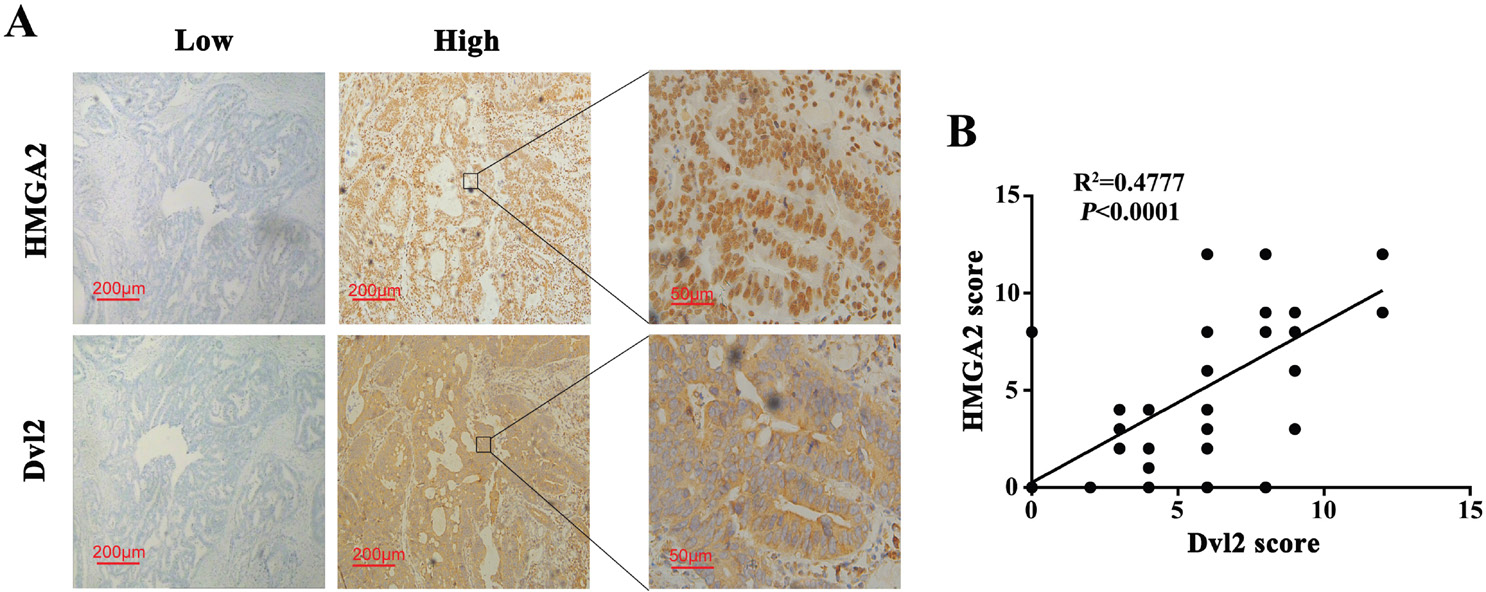
Dvl2 expression is positively correlates with HMGA2 expression in human CRC tissues (**A**) HMGA2 and Dvl2 expressions in CRC samples were determined by immunohistochemical staining. Representative images of HMGA2 and Dvl2 in CRC tissues were shown. (**B**) The correlation between Dvl2 expression and HMGA2 expression was analyzed in CRC tissues (*N* = 61, R2 = 0.4777, *P* < 0.0001). Each dot presented one case.

To explore whether Dvl2 was correlated with HMGA2 expression, we assessed HMGA2 and Dvl2 protein levels in the 61 patients who received first-line 5-FU-based adjuvant chemotherapy by immunohistochemical analysis. We found a significant positive correlation between HMGA2 and Dvl2 expressions (R2 = 0.4777, *P* < 0.0001, Figure 7A and 7B). Taken together, these data demonstrated that HMGA2/Dvl2 played an important role in chemoresistance to 5-FU in CRC, which would indicate a high risk of a poor clinical outcome.

## DISCUSSION

Since the 1960s, 5-FU has become the standard treatment for CRC. However, resistance to 5-FU treatment is one of the major causes of chemotherapy failure in advanced CRC. Among the complex mechanisms accounting for chemoresistance, anti-apoptosis is an important factor [17–19], and hyperactivation of the Wnt pathway is one of the major causes of increased cell proliferation and the induction of drug resistance [20, 21]. Specifically, signaling pathways such as the Notch, Wnt, and nuclear factor-κB pathways play major roles in mediating self-renewal, which also contribute to drug resistance [22]. Therefore, therapeutically targeting these pathways to enhance the efficacy of conventional chemotherapy is an attractive strategy for further improvement of the treatment response in advanced CRC patients.

HMGA2, a member of the non-histone chromosomal protein family, is correlated with RNA processing, transcriptional regulation and chromatin remodeling [23]. Induced HMGA2 expression directly interferes with cell differentiation and transformation, cell proliferation, metastasis and epithelial mesenchymal transition (EMT) in many malignant tumors [24–26]. Recently, Wu et al. [27] reported that high miR-204 expression reduced cell sensitivity to 5-FU-based treatment through HMGA2 in CRC. However, the exact mechanism underlying the role of HMGA2 in regulating 5-FU chemoresistance in CRC is still not well understood. Our results demonstrate that overexpression of HMGA2 enhances chemoresistance to 5-FU both *in vitro* and *in vivo*.

Based on bioinformatic analysis, we hypothesized that Wnt signaling is involved in the HMGA2-mediated chemoresistance of CRC cells. Abnormal activation of Wnt/β-catenin signaling is one of cellular characteristics involved in the development and drug resistance of a series of cancers. In CRCs, a hallmark of Wnt activation is APC gene mutations [28, 29] that subsequently lead to the nuclear localization of β-catenin. Nuclear β-catenin functions as a transcription factor together with the TCF (T-cell factor)/LEF (lymphocyte enhancing factor) family to trigger the expression of downstream target genes related to a vast array of biological behaviors, including cell proliferation, differentiation, apoptosis, epithelial-mesenchymal transition, polarity establishment and stem cell self-renewal[30–33]. However, high expression of Dvl2 can also efficiently activate Wnt/β-catenin signaling independent of Wnt stimulation [34, 35]. The dishevelled family was first identified based on a phenotype of disorientation in the body and wing hairs of Drosophila [36]. Three Dvl homologues (Dvl1, 2, 3) are present in mammals. Deletion of Dvl2 reduces intestinal length and inhibits neoplasia formation in the APCMin mouse model of CRC [37]. In the canonical Wnt/β-catenin pathway, Dvls act as mediators linking together Wnt receptors and downstream effectors [38]. Elevated Dvl2 expression can aid in the release of β-catenin from the cytosolic Axin/GSK-3/APC complex through inhibiting the activity of GSK3β [39]. In addition, Gan et al. [40] revealed that Dvl2 bound to c-Jun to mediate the formation of the Dvl/c-Jun/β-catenin/TCF functional complex, leading to stabilization of the β-catenin-TCF interaction. All of these results reveal a mechanism by which Dvl2 ultimately triggers the expression of Wnt/β-catenin target genes. Using ChIP and dual luciferase reporter gene assays (truncation and mutation), we demonstrated that HMGA2 promoted Dvl2 expression via transcriptional modification, leading to increased activation of the Wnt/β-catenin pathway. In addition, Dvl2 was shown to be required for the HMGA2-mediated chemoresistance of CRC cells. Furthermore, HMGA2 and Dvl2 expression were positively correlated in CRC patients.

HMGA2 is an architectural transcription factor that regulates gene expressions through protein-DNA and protein-protein interactions [41]. Additional alterations of target genes or proteins may be required for HMGA2-mediated chemoresistance to occur. The characterization of the functional and mechanistic relationship between HMGA2 and other genetic events should be explored in the future. In addition, 5-FU is often used for the treatment of other cancer types; thus, further studies will determine whether suppression of HMGA2 could inhibit drug resistance to 5-FU in other tumors.

In conclusion, our data demonstrate that overexpression of HMGA2 enhances resistance to 5-FU-based chemotherapy in CRC. In addition, HMGA2-mediated chemoresistance is at least partly mediated via upregulation of Dvl2 and subsequent activation of the Wnt/β-catenin pathway. Based on the foundation of this work and previous studies, the HMGA2/Dvl2 axis is of considerable therapeutic significance, and methods aimed at suppressing the HMGA2/Dvl2 axis may provide a new modulation strategy for overcoming chemoresistance in CRC.

## MATERIALS AND METHODS

### Reagents and materials

Cell-culture reagents were purchased from Gibco (Grand Island, USA). Lipofectamine and TRIzol were obtained from Invitrogen (Carlsbad, USA). Secondary antibodies for Western blotting were purchased from Li-COR, USA. The siRNA targeting Dvl2 was obtained from Gene Pharma (Shanghai, China).

### Cell cultures

SW620, HCT116, RKO and HT29 cells were purchased from the American Type Culture Collection (ATCC) and cultured in RPMI 1640 supplemented with 10% fetal bovine serum (Invitrogen, USA). HEK293T cells were cultured in DMEM supplemented with 10% fetal bovine serum. All cell lines were maintained in a 5% CO2 atmosphere.

### Cell viability assay

Briefly, 96-well plates were seeded with 5,000 cells/well, and the cells were incubated in 1% serum medium with 5-FU for 48 h. The cells were subsequently stained for 4h with MTT [(3-(4,5)-dimethylthiazol-2-yl)-2,5-diphenyltetrazolium bromide] (Sigma, USA), and the OD at 570 nm was read after dissolving in DMSO. Cell viability was calculated as a ratio of OD values of drug-treated samples to those of controls.

### Western blotting

Cells were lysed with RIPA buffer and boiled for 5 minutes. The obtained protein lysates were resolved by SDS-polyacrylamide gel electrophoresis (SDS-PAGE) and transferred to nitrocellulose filter membranes. Then, the following primary antibodies and dilutions were applied: Dvl2 (1:1000, GeneTex), Cleaved PARP (1:1000, Cell Signaling Technology), Cleaved-Caspase-9 (1:1000, Cell Signaling Technology), Cleaved-Caspase-3 (1:1000, Cell Signaling Technology), CD44 (1:1000, Cell Signaling Technology), β-catenin (1:1000, Cell Signaling Technology), c-Myc (1:1000, Cell Signaling Technology), cyclin D1 (1:500, Cell Signaling Technology), MMP7 (1:5000, Abcam), and GAPDH (1:5000, Santa Cruz).

### RNA isolation and quantitative RT-PCR analysis

Total RNA was extracted using the TRIzol method. Then, RT-PCR was performed using reagents from TaKaRa according to the manufacturer’s manual. Quantitative PCR amplifications were performed with SYBR Green (TaKaRa, Japan). The gene transcripts were normalized to the transcript levels of the housekeeping gene GAPDH. The primers employed for these assays were listed in Supplementary Table 1.

### Luciferase assay

Cells were transfected with firefly luciferase reporter constructs and the control Renilla luciferase reporter pRL-TK using Lipofectamine (Invitrogen, USA). After treatment, the cells were lysed with the cell lysis buffer provided in the dual-luciferase reporter assay kit (Promega, USA). Luciferase activity was then measured according to the manufacturer’s instructions.

### Chromatin immunoprecipitation (ChIP)

Chromatin was cross-linked using 1% formaldehyde for 15 min and sonicated to obtain DNA fragments of 200–500 bp. After centrifugation, the supernatants were subjected to immunoprecipitation overnight at 4°C with antibodies against HMGA2 or normal rabbit IgG. ProteinA-Agarose beads were used to isolate the chromatin-antibody complexes. The crosslinking was reversed and the precipitated DNA fragments were purified and analyzed by PCR. The primers used were used as follows: forward 5′-ACTCCTGCGGGTCAGAGTT-3′ and reverse 5′-GCTGCCTTCAGCAACGGTC-3′ (site 1), forward 5′-TACCTGGAGGAAGCTCGCG-3′ and reverse 5′-AGCGCGGACAGGACTGACCCA-3′ (site 2).

### Immunohistochemistry

A total of 61 human CRC samples were collected at the Sanmen People’s Hospital and after informed consent was obtained from all patients. Immunohistochemistry was performed using an Envision Detection System (DAKO, USA) according to the manufacturer’s instructions. The following antibodies were employed in these assays: HMGA2 (1:25, Abcam) and Dvl2 (1:200, GeneTex). Each specimen was scored as follows: 0, negative or < 5% positive cells; 1, 5–25% positive cells; 2, 26–50% positive cells; 3, 51–75% positive cells; 4, > 75% positive cells. The staining intensities were graded from 0-3: 0 for no staining, 1 for weak staining, 2 for moderate staining, 3 for strong staining. Subsequently, two independent investigators assessed and confirmed the staining results. The percentage of positivity among the tumor cells and the staining intensities were then multiplied to generate the IHC scores, which were graded as corresponding to low expression (score 0∼6) or high expression (score 8∼12). Cases with a discrepancy in scores were discussed to obtain a consensus. The study was approved by the Zhejiang University Institutional Reiew Boards.

### Plate colony formation assay

1 × 103 cells per well of a six-well plate were treated with a single dose of 5-FU for 1 week. Resistant clones were fixed with 4% paraformaldehyde and stained with 0.2% crystal violet and counted. Crystal violet retained in the cells was quantified by solubilization with 0.5% acetic acid and measurement of optical density at 592 nm.

### Animal experiments

BALB/c-nu/nu mice (4 weeks old, male) were purchased from the Shanghai Laboratory Animal Center. Cells were harvested and suspended in PBS. HMGA2-overexpressing SW620 cells or their corresponding control cells (1 × 107 cells; *n* = 5 mice in each of group) in 0.1 ml of PBS were injected subcutaneously into the flanks of mice (5-week-old). One week after injection, 5-FU (40 mg/kg) or carrier was administered via intraperitoneal injection every three days. Tumor volumes were measured using digital calipers every three days from the beginning of treatment to the end point. Tumor volume was calculated using the following formula: π/6 × (length) × (width)2. All procedures for animal study were approved by the Animal Care and Use Committee of Zhejiang University.

### Statistical analysis

A database was created and transferred to SPSS 22.0 and GraphPad Prism 6.0 for Windows. Statistical data analysis was performed using the two-tailed Student’s *t*-test, chi-squared test, or one-way ANOVA. The results are presented as the mean ± SD of three separate experiments. A value of *P* < 0.05 was considered statistically significant. Spearman’s test was employed to analyze the correlations.

## SUPPLEMENTARY MATERIALS TABLES






