# Supplementary material for: HMGA2 enhances 5-fluorouracil chemoresistance in colorectal cancer via the Dvl2/Wnt pathway
Source: Oncotarget. 2018 Jan 10;9(11):9963–74. doi: 10.18632/oncotarget.24133 (PMC5839414; doi:10.18632/oncotarget.24133)
Supplement: Supplementary file 1 [file oncotarget-09-9963-s001.pdf]

# HMGA2 enhances 5-fluorouracil chemoresistance in colorectal cancer via the Dvl2/Wnt pathway

## SUPPLEMENTARY MATERIALS

**Supplementary Table 1: Primers used for quantitative RT-PCR analysis**

| Name | Sequence (5′ to 3′) |
|---|---|
| GAPDH-forward | GAAGGCTGGGGCTCATTGA |
| GAPDH-reverse | GCTGATGATCTTGAGGCTGTTGT |
| c-myc-forward | GCTGGACCAGATGTATGTCCC |
| c-myc-reverse | ATCATTTCCATGACGGCCTGT |
| CD44-forward | AGCCCATGTTGTAGCAAACC |
| CD44-reverse | TGAGGTACAGGCCCTCTGAT |
| Dvl2-forward | GAGGAAGAGACTCCCTACCTG |
| Dvl2-reverse | CGGGCGTTGTCATCTGAAAT |
| FZD7-forward | GTGCCAACGGCCTGATGTA |
| FZD7-reverse | AGGTGAGAACGGTAAAGAGCG |
| MMP7-forward | ATGTGGAGTGCCAGATGTTGC |
| MMP7-reverse | AGCAGTTCCCCATACAACTTTC |
| Smad6-forward | GCTACCAACTCCCTCATCACT |
| Smad6-reverse | CGTACACCGCATAGAGGCG |
| Smad7-forward | TTCCTCCGCTGAAACAGGG |
| Smad7-reverse | CCTCCCAGTATGCCACCAC |
| IGFBP6-forward | AGGAGTGCGGGGTCTACAC |
| IGFBP6-reverse | CTCTGCGGTTCACATCCTGT |
| TNFSF9-forward | GGCTGGAGTCTACTATGTCTTCT |
| TNFSF9-reverse | ACCTCGGTGAAGGGAGTCC |
| β-catenin-forward | GAGCCTGCCATCTGTGCTCT |
| β-catenin -reverse | ACGCAAAGGTGCATGATTTG |
| cyclin D1-forward | TCCTCTCCAAAATGCCAGAG |
| cyclin D1-reverse | GGCGGATTGGAAATGAACTT |

**Supplementary Table 2: Gene sets enriched in phenotype positive correlation with profile.** See_ Supplementary_Table _2

**Supplementary Table 3: Table Gene sets enriched in phenotype positive correlation with profile plain text format.** See_Supplementary_Table _3
